# The Eye is Listening: Music-Induced Arousal and Individual Differences Predict Pupillary Responses

**DOI:** 10.3389/fnhum.2015.00619

**Published:** 2015-11-10

**Authors:** Bruno Gingras, Manuela M. Marin, Estela Puig-Waldmüller, W. T. Fitch

**Affiliations:** ^1^Institute of Psychology, University of InnsbruckInnsbruck, Austria; ^2^Department of Basic Psychological Research and Research Methods, University of ViennaVienna, Austria; ^3^Department of Cognitive Biology, University of ViennaVienna, Austria

**Keywords:** music, emotion, arousal, individual differences, pupillometry

## Abstract

Pupillary responses are a well-known indicator of emotional arousal but have not yet been systematically investigated in response to music. Here, we measured pupillary dilations evoked by short musical excerpts normalized for intensity and selected for their stylistic uniformity. Thirty participants (15 females) provided subjective ratings of music-induced felt arousal, tension, pleasantness, and familiarity for 80 classical music excerpts. The pupillary responses evoked by these excerpts were measured in another thirty participants (15 females). We probed the role of listener-specific characteristics such as mood, stress reactivity, self-reported role of music in life, liking for the selected excerpts, as well as of subjective responses to music, in pupillary responses. Linear mixed model analyses showed that a greater role of music in life was associated with larger dilations, and that larger dilations were also predicted for excerpts rated as more arousing or tense. However, an interaction between arousal and liking for the excerpts suggested that pupillary responses were modulated less strongly by arousal when the excerpts were particularly liked. An analogous interaction was observed between tension and liking. Additionally, males exhibited larger dilations than females. Overall, these findings suggest a complex interplay between bottom-up and top-down influences on pupillary responses to music.

## Introduction

Music is a powerful elicitor of emotions (Blood et al., 1999), and there is cumulative empirical evidence that emotions induced by music share many components typical of emotions induced by other types of sensory stimuli (Scherer, 2004; Juslin and Västfjäll, 2008). Musical emotions can be evoked by various mechanisms that vary in their degree of relatedness to acoustical and musical properties (Juslin and Västfjäll, 2008). Emotion-inducing mechanisms such as evaluative conditioning or episodic memory do not depend on the music’s acoustical and musical features but rather on the type of emotions induced by real-life events that were coupled with the experience of music (Juslin and Västfjäll, 2008). Conversely, emotion induction by emotional contagion is a mechanism that largely depends on the musical signal itself. Musical emotions are perceived, trigger physiological responses, and concurrently induce emotions in the listener. Musically induced emotions are conveyed by two types of cues, comprising basic acoustic cues, such as those related to intensity (Juslin and Laukka, 2003; Ilie and Thompson, 2006), timbre (Scherer and Oshinsky, 1977; Hailstone et al., 2009), pitch, and tempo (Hevner, 1937; Ilie and Thompson, 2006), and culturally determined cues associated with a specific musical system (Meyer, 1956). In particular, subjective arousal induced by music has been associated with intensity, tempo, and spectral parameters such as spectral flux and spectral entropy (Gingras et al., 2014).

There is a growing body of research on psychophysiological indices of music-induced emotions, such as skin conductance, heart rate, and facial electromyography (e.g., Gomez and Danuser, 2004; Rickard, 2004; Khalfa et al., 2008; Lundqvist et al., 2009). However, very few studies have been published on pupillary responses in relation to music, as pointed out in a recent review (Hodges, 2010, Table 11.2), even though the influence of emotional processing on pupillary responses has been clearly established with both pictures (Steinhauer et al., 1983; Bradley et al., 2008) and environmental sounds (Partala and Surakka, 2003). Considering that music is recognized as a potent inducer of emotions in everyday life (Sloboda, 2010), especially with respect to emotional arousal (Khalfa et al., 2002; Gomez and Danuser, 2004; van den Bosch et al., 2013), it would seem to be an ideal stimulus for investigating pupillary responses. In contrast to visual stimuli, auditory stimuli present an important advantage for the study of pupillary responses, in that methodological problems related to color, luminance, and contrast are circumvented (Hess and Petrovich, 1987). Moreover, pupillometry is less invasive than other psychophysiological measurements such as electrodermal activity, respiratory patterns, and heart rate. An additional benefit of using pupillary responses to investigate music-induced emotions is that pupillary dilations in response to external stimuli or mental events cannot be voluntarily suppressed (Loewenfeld, 1993).

Variations in pupil size are considered to be a reliable indicator of autonomic nervous system (ANS) activity (Andreassi, 1995). Importantly, pupil diameter is affected not only by changes in ambient light (the pupillary light reflex), but also by non-visual stimuli as well as cognitive load and affective processing (Goldwater, 1972; Laeng et al., 2012). Indeed, pupillary dilation has been observed in response to emotionally relevant visual (Hess and Polt, 1960; Hess et al., 1965) or auditory (Dabbs, 1997; Partala and Surakka, 2003) stimuli, as well as to increased working memory (Kahneman and Beatty, 1966), or executive loads (Hess and Polt, 1964; Ahern and Beatty, 1979).

Changes in pupil diameter are controlled by two muscles, the pupil dilator muscle and the pupillary sphincter. The activity of the dilator muscle is mediated by the sympathetic pathway, whereas the pupillary sphincter is under control of the parasympathetic system, mediated by the Edinger–Westphal complex of the oculomotor nucleus (Steinhauer et al., 2004). Although pupillary responses to increased cognitive load are primarily mediated by the parasympathetic system (Steinhauer et al., 2004), pupil dilations observed in response to emotional stimuli may involve activation of the dilator muscle by the sympathetic system (Bradley et al., 2008).

Pupil diameter can react to stimulation in as little as 0.2 s (Lowenstein and Loewenfeld, 1962). Although changes in illumination can induce pupillary dilations corresponding to an increase of up to 4 mm, changes that are cognitively driven are more modest and are rarely greater than 0.5 mm (Beatty and Lucero-Wagoner, 2000), corresponding to a size increase of approximately 20%. Additionally, pupillary responses seem to be affected by the interaction between emotional influences and cognitive load. An earlier study by Stanners et al. (1979), which manipulated both cognitive load and arousal, concluded that arousal affected pupillary responses only when cognitive load was low. This finding is congruent with the fact that studies reporting effects of emotion in terms of sympathetic activation employed low cognitive-load tasks such as passive viewing or listening (Partala and Surakka, 2003).

To our knowledge, the earliest published study on music-induced pupillary responses is that of Slaughter (1954), which used a subjective, observational methodology to determine that stimulative music led to pupil dilation, while sedative music induced pupil constriction. Mudd et al. (1990) reported an association between pupil responses to music and noise excerpts and preference ratings. Using stimuli from the International Affective Digitized Sounds (IADS) database (Bradley and Lang, 1999), Partala and Surakka (2003) showed that pupil size was larger during emotionally negative or positive stimuli than during neutral sounds. Roy et al. (2009) observed that the startle eye blink reflex occurred faster and attained a larger amplitude for unpleasant music than for pleasant music.

In the present study, we investigated pupillary responses to music within the context of the multicomponent model of musical responses proposed by Hargreaves et al. (2005). In their reciprocal feedback model, Hargreaves et al. (2005) describe three main determinants of emotional responses to a musical stimulus: the music, the listening situation and the listener. Here, we focused more specifically on the combined influence of affective characteristics specific to a musical excerpt (such as arousal potential) and traits associated with a particular listener (such as personality traits or orientation to music) on pupillary responses to music. Modern statistical approaches such as linear mixed models (Laird and Ware, 1982) allow the estimation of such *excerpt-specific* and *listener-specific* effects in a single statistical model. In order to investigate both effects, we collected pupillary responses to a large set of short musical stimuli for which subjective emotion and familiarity ratings had previously been collected (Gingras et al., 2014), and invited participants to complete a series of questionnaires to assess their personal characteristics.

Because gender effects for psychophysiological arousal measures have been previously reported, with females displaying stronger responses to highly arousing stimuli (Bradley et al., 2001; Partala and Surakka, 2003; Nater et al., 2006), we predicted that females would show greater pupillary dilation in response to highly arousing stimuli. Since Nater et al. (2006) proposed that stress reactivity, for which females generally report higher values, may explain these gender effects, we also assessed participants’ stress reactivity. Moreover, stress reactivity has also been discussed in relation to emotion regulation and physiological functioning during music listening (Thoma et al., 2012). Here, stress reactivity was evaluated using the German Stress Reactivity Scale (SRS), which is based on the assumption that four personality characteristics underlie stress reactivity: high intrusiveness, low self-efficacy, high arousability of the central nervous system, and high negative affectivity (Schulz et al., 2005).

Temporary mood states may affect emotion processing (Eerola and Vuoskoski, 2010; Schmid and Schmid Mast, 2010; Vuoskoski and Eerola, 2011; Cummings and Rennels, 2014), thus participants’ mood was assessed prior to the experiment using the multidimensional mood questionnaire (Mehrdimensionaler Befindlichkeitsfragebogen, MDBF; Steyer et al., 1997). Furthermore, we obtained overall liking ratings for the music excerpts used in the experiment to account for individual differences regarding preferences for musical styles (Kreutz et al., 2008) as well as for the link between physiological arousal and liking (Berlyne, 1971; Schäfer and Sedlmeier, 2010). Finally, participants were asked to estimate the frequency with which they experienced emotions while listening to the excerpts during the experiment, and more generally to assess the role of music in their lives as a broad measure of musical engagement, i.e., involvement with and interest in music.

Both Partala and Surakka (2003) and Bradley et al. (2008) suggested, on the basis of their results, that pupil dilation was determined mostly by emotional arousal. Bradley et al. (2008) additionally reported a strong concordance between pupil dilation and skin conductance, another measure of physiological arousal. Thus, we hypothesized that subjective arousal ratings should predict pupillary responses to musical stimuli, with a larger pupil dilation for excerpts judged as highly arousing. However, there was a potential confound in both studies cited above: neutral stimuli were subjectively rated as significantly less arousing than either positively or negatively valenced stimuli, and therefore effects of valence could not be disentangled from those of arousal. Here, we addressed these issues by also including neutrally valenced stimuli with a broad range of arousal ratings.

Previous studies on pupillary responses induced by auditory stimuli did not consider emotion models incorporating other affective dimensions such as tension, which have been argued to be more suitable to music emotion research (Schimmack and Grob, 2000; Schimmack and Reisenzein, 2002). Here, we used a three-dimensional emotion model (pleasantness, arousal, and tension) to predict pupillary responses. To do so, we invited a second group of participants to rate the music excerpts for arousal (calm versus aroused), tension (relaxed versus tense), and pleasantness (unpleasant versus pleasant), following Wundt’s (1896) model, and compared these ratings with the pupillary responses observed in response to the same excerpts, but in a different group of participants.

Careful attention was paid to the selection of musical stimuli. All music excerpts used in the present study were obtained from a selection of Romantic piano trios, a relatively unfamiliar musical genre characterized by a high stylistic and timbral uniformity. We used a set of excerpts matched for timbre and compositional style because the relationship between emotional ratings and acoustic cues has been shown to be partly genre-specific (Eerola, 2011). Additionally, to minimize potential confounds due to effects of familiarity on emotion ratings (Witvliet and Vrana, 2007; Marin and Leder, 2013; van den Bosch et al., 2013), we chose a musical style with which most listeners are likely to be unaccustomed but which is still rooted in familiar Western major–minor tonality. The use of recordings of actual performances ensured that listeners had access to any ecologically relevant acoustic information that may play a role in eliciting emotional responses.

Finally, we considered the possible effect of sound intensity on pupil dilation. Stelmack and Siddle (1982) observed similar pupillary responses for three intensity levels (60, 75, and 90 dB) of a 1000-Hz pure tone and concluded that tone intensity had no reliable effect on the amplitude of pupillary dilation. However, other researchers found that louder pure tones (Nunnally et al., 1967; Hirano et al., 1994) or broadband noise (Antikainen and Niemi, 1983) led to larger pupillary dilations. Because sound intensity has been linked to subjective arousal (Scherer, 1989; Ilie and Thompson, 2006) and to measurements of physiological arousal such as skin conductance (Gomez and Danuser, 2007), we used amplitude-normalized excerpts for this study and verified that our excerpts were adequately matched for perceptual loudness as well (Gingras et al., 2014). Amplitude normalization is a procedure routinely used in psychoacoustic research (e.g., Bigand et al., 2011), and specifically to control for differences in arousal induction modulated by sound intensity (e.g., Roy et al., 2009).

## Materials and Methods

### Stimuli

Eighty-four 6-s excerpts were selected from commercial recordings (lossless audio) of piano trios from the Romantic period, corresponding to the early to middle 19th-century. All three instruments of the trio (piano, violin, and cello) could be heard at least once during each excerpt. To avoid intra-opus familiarity effects (Krumhansl, 1995), only one excerpt per movement was chosen. Following Hevner’s (1935) recommendation, only excerpts with a uniform emotional expression were selected. Linear fade-in and fade-out were applied to the first and last 22 ms of each excerpt. A list of the excerpts is provided in the Appendix in the Supplementary Material.

Excerpts were globally normalized at the mean intensity level of all original excerpts, such that the average intensity was the same for all excerpts (details are provided in the “Materials and Methods” section of Gingras et al., 2014). Because the normalization was done on the mean intensity levels computed over the entire excerpts, intensity contours were preserved intact for each excerpt. Four excerpts with mean familiarity ratings over 4 (middle of scale) were excluded from this analysis, leaving 80 excerpts. Note that, whereas participants who rated the excerpts for arousal and valence in Gingras et al. (2014) heard all 84 excerpts, the two groups of participants recruited for this study (see below) heard only the 80 excerpts selected as described above.

### Participants

Thirty German-speaking psychology students (15 females, mean age = 23.1 years, *SD* = 2.6, range: 19–30) rated the musical excerpts for arousal, tension, valence, and familiarity. Another thirty German-speaking participants, for the most part university students (15 females, mean age = 26.1 years, *SD* = 5.8, range: 19–39), participated in the pupillary response experiment. All participants for both experiments had less than 3 years of musical training, were not musically active at the time of the experiment, and reported normal hearing and no history of hearing disorders. Participants in the pupillary response experiment had normal or corrected-to-normal vision. All experiments conformed to the institutional guidelines of the University of Vienna for experiments with human subjects. Written informed consent was given by all participants who could withdraw at any time during the experiment without further consequences. All experimental data were collected between November 2012 and July 2013.

### Procedure for the Subjective Rating Experiment

The procedure for the subjective rating experiment was identical to the procedure described in Gingras et al. (2014), except that participants also rated the excerpts for tension. Briefly, participants first filled out the MDBF mood questionnaire (short form version A) and were instructed to rate their familiarity with the musical excerpts, as well as their felt arousal, felt tension, and felt pleasantness, using 7-point scales. The scales ranged from “very unfamiliar” to “very familiar” for familiarity, “very calm” to “very aroused” for arousal, “very relaxed” to “very tense” for tension, and “very unpleasant” to “very pleasant” for pleasantness. In order to familiarize participants with the procedure, they first practiced with three excerpts not included in the actual stimulus set and were then exposed to all 80 excerpts from the stimulus set. The order of presentation of the excerpts was randomized. Ratings were entered on the computer (by clicking on ordered icons on the screen corresponding to the scale ratings) only once the entire excerpt was played. After all ratings were entered, there was a 5-s delay before the next excerpt began playing. Excerpts were presented using an E-MU 0204 USB audio interface (E-MU, Scotts Valley, CA, USA), at a fixed intensity level, on Sennheiser HD 380 headphones. Stimulus presentation and ratings collection were controlled using a custom MATLAB interface. The entire experiment lasted approximately 45 min.

### Procedure for the Pupillary Response Experiment

The EyeLink 1000 head-supported infrared optical eye-tracking system (SR Research, Ottawa, ON, Canada), which includes a 1000-Hz infrared camera, illuminator, and proprietary software running on a custom workstation, was used to collect pupil data. The screen used for the experiment was a Samsung SyncMaster 2233 (21.5 inches, 60 Hz refresh rate), with a resolution of 1680 × 1050 pixels. The background color of the screen was gray, RGB (150,150,150), following Kuchinke et al. (2007). The computer was an Apple Mac Mini 4.1, with an Intel Core Duo 2 2.4 GHz processor, running on the Mac OS X 10.6.7 operating system. Musical excerpts were played using an Edirol FA-66 FireWire Audio Capture audio interface (Roland, Shizuoka, Japan), at a fixed intensity level, on Sennheiser HD 280 headphones. The mean intensity across all excerpts was 70 dB SPL, based on audiometric measurements taken at the headphones using a Voltcraft SL-400 decibel meter that was calibrated immediately prior to usage. The stimuli were presented and the experiment was controlled using Psychtoolbox-3.0.9 (Brainard, 1997; Pelli, 1997; Cornelissen et al., 2002) running on MATLAB R2010a (Mathworks, Natick, MA, USA).

Participants first signed the informed consent form and filled out the MDBF mood questionnaire. They were then seated in a comfortable chair with their head stabilized in a chin rest, facing the computer monitor at a distance of 60 cm, in a quiet, moderately lit room (ambient light levels of 200 lux as measured just below the forehead support using an X-Rite i1Pro lux meter). A randomized target order 5-point (HV5) calibration routine was performed (5-point calibration was deemed sufficient since pupil diameter was the only measurement of interest and participants were asked to continuously fixate the area corresponding to the center of the screen), followed by a separate validation using the EyeLink 1000 software. Participants were asked not to move their head during the experiment and to look at the fixation cross located at the center of the screen and try to avoid blinking when it was displayed (they were shown an image of the cross). Participants were also told that they could blink or close their eyes when a “smiley face” was shown on the screen in-between trials. The cross color was dark gray (RGB: 75,75,75). The size of the fixation cross was 168 × 168 pixels, corresponding to 4.5° of visual angle at a viewing distance of 60 cm. The smiley face was the same color as the cross and approximately the same size.

As with the rating experiment, participants first practiced with three excerpts not included in the actual stimulus set and were then exposed to all 80 excerpts from the stimulus set. The order of presentation of the excerpts was randomized. For each excerpt, the fixation cross was first shown for 2 s, then the music played for 6 s, then the cross was displayed for another 2 s, for a total of 10 s of recording of the pupillary response per trial. Similar to the rating procedure, there was a 5-s delay between excerpts, indicated by a “smiley face” displayed on the screen, during which participants could close their eyes or blink. Four seconds after the end of a trial (1 s before the cross indicating the beginning of the next trial would appear), a soft “beep” sound (a 400 Hz pure tone played for 0.1 s) was played to indicate that the participants should prepare to open their eyes and look at the fixation cross. After 40 stimuli (midway through the experiment), participants were allowed to take a pause. Upon resuming the experiment, calibration correction was performed (complete calibration was performed if necessary).

Once all excerpts had been played, participants were invited to fill in a post-experiment paper questionnaire about their socio-demographic background and musical interests. This questionnaire included three questions, all on a 7-point scale, about the role that music plays in their life (ranging from “no role” to “a very important role”; the German acceptation of the term refers to the general importance of music in participants’ lives), their general liking for the excerpts presented in the experiment (ranging from “not at all” to “very much”) and the frequency with which they felt emotions during the experiment (ranging from “never” to “very often”). Participants also completed the SRS (Schulz et al., 2005), in which each item describes a potentially stressful situation with three answer options representing possible stress responses. Finally, participants were paid 5 Euros for their participation, thanked, and debriefed. The entire experiment lasted approximately 30 min.

### Data Analysis

The left eye’s pupil diameter and gaze coordinates were sampled at 1,000 Hz with an average spatial resolution of 20 min arc (range across participants: 11–39 min arc). Pupil diameter is measured in arbitrary units which are linear in true diameter (Einhäuser et al., 2008). Gaze coordinates were also recorded in order to track the gaze position and exclude samples for which the participants did not fixate the screen area corresponding to the center cross.

Blinks were identified by the proprietary algorithm of the Eyelink 1000 eye-tracking system, using default settings. Data samples from 50 ms before the beginning of blinks to 50 ms after the end of blinks were discarded to exclude pre- and post-blink artifacts (1.7% of all samples; range: 0.1–5.0% per participant). In addition, given that pupil size estimation is less accurate when participants are not fixating the center of the screen (Gagl et al., 2011), all samples for which the screen coordinates of the gaze were outside a circle centered on the fixation cross and with diameter equal to the size of the cross (168 pixels) were excluded (0.3% of all samples; range: 0.0–4.7% per participant). Following Einhäuser et al. (2008), all discarded samples were treated as missing data rather than interpolated (Einhäuser et al., 2008 obtained very similar results with both methods). Trials during which participants blinked or did not fixate the center cross for more than 15% of the total trial duration were excluded. A total of 50 of 2400 trials (2.1%) were thus excluded (range: 0.0–8.8% per participant).

Frequency responses in pupil size variation that occur at rates faster than 2 Hz are considered to be noise (Richer and Beatty, 1985; Privitera and Stark, 2006). Accordingly, pupil diameter data were low-passed using a fourth-order Butterworth filter with a cutoff frequency of 4 Hz. The baseline pupil diameter was measured as the average pupil diameter for a period of 200 ms immediately preceding the stimulus onset. Baseline-corrected pupil diameters were computed by subtracting the baseline pupil diameter from the raw pupil diameter after stimulus onset. To allow for comparisons between participants and to correct for possible tonic changes in pupil diameter over the course of the experiment, raw pupil diameters were converted into relative pupil diameter by expressing them as a proportional difference from the baseline diameter (van Rijn et al., 2012).

All data analyses were conducted in MATLAB R2012b (Mathworks, Natick, MA, USA), except for the linear mixed model analysis which was implemented in R 3.1.1 (R Core Team, 2015) using the *lmer* function from package lme4 to build the models (Bates et al., 2015), the *fitLMER* function from package LMERConvenienceFunctions to select the best-fitting models (Tremblay and Ransijn, 2015) and the *Anova* function from package car to obtain significance tests (Fox and Weisberg, 2011). Statistical power estimates were computed with G^∗^Power 3.1.9.2 (Faul et al., 2007).

## Results

### Mood Questionnaire

The MDBF mood questionnaire includes subscales for positive/negative mood, alertness/fatigue, and quietude/disquietude. Subscale scores were analyzed using a MANOVA design, with experimental group (subjective ratings versus pupillary response) as a between-subject factor. Except for a marginal tendency for positive/negative mood scores to be lower for the subjective rating group, *F*(1,58) = 3.45, *p* = 0.069, no significant differences were observed between the two groups on the subscales (all other *p*-values >0.3).

### Subjective Ratings of the excerpts

#### Familiarity

The overall mean familiarity rating for the 80 excerpts was 2.76 (range: 2.00–3.73, *SD* = 0.41) on a 7-point scale, which is slightly lower than the ratings obtained on the same excerpts in Gingras et al. (2014) and suggests that none of the excerpts sounded very familiar to the participants.

#### Arousal, Tension, and Pleasantness

**FIGURE 1:**
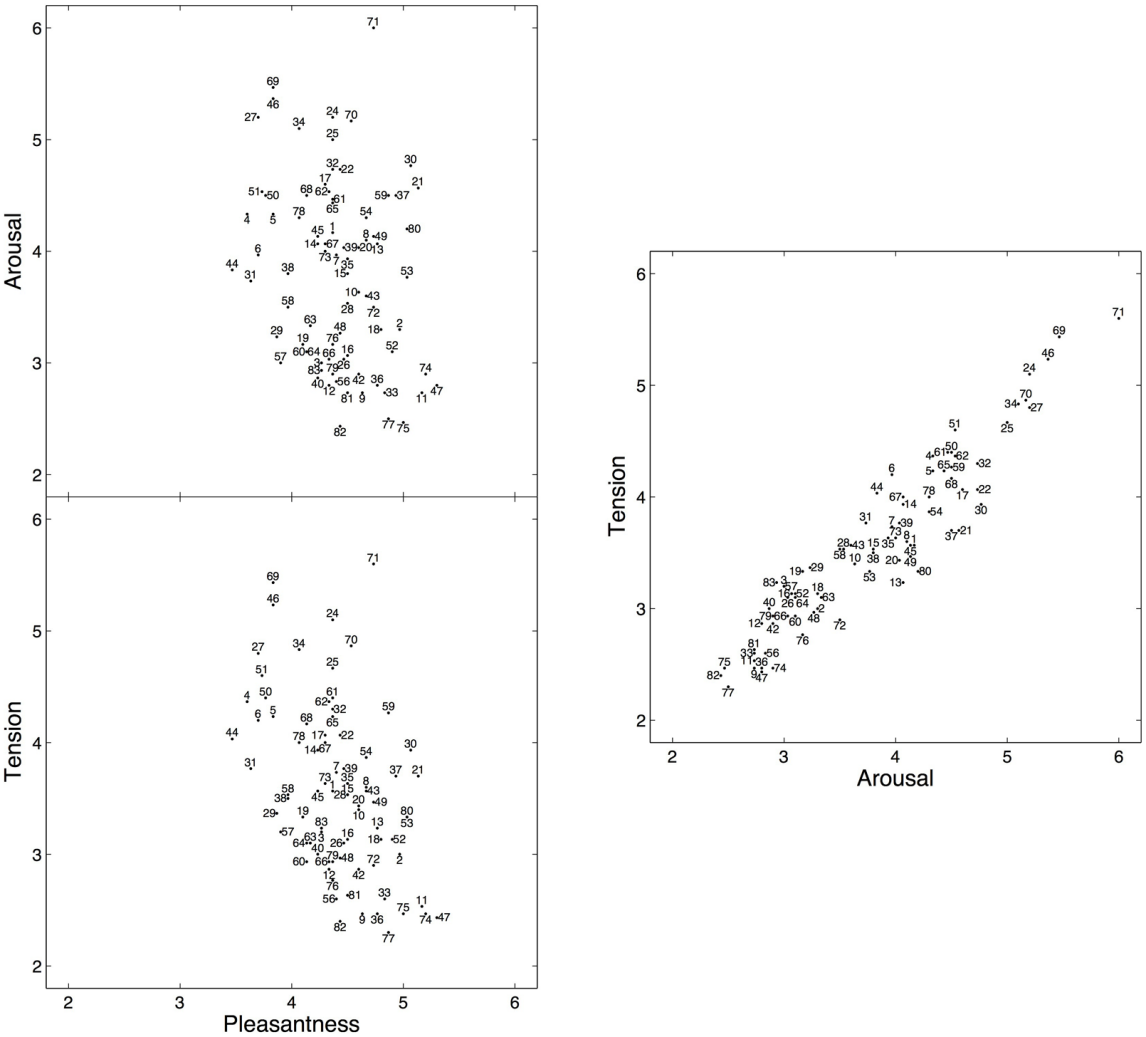
**Mean subjective arousal, tension, and pleasantness ratings for 80 six-second excerpts selected from Romantic piano trios**. The numbers identify the excerpts (for a complete listing of the excerpts, see Appendix in the Supplementary Material). The full scale for all three ratings ranged from 1 to 7, but a restricted range is displayed here to facilitate viewing.

To evaluate whether participants rated the excerpts in a consistent manner, inter-rater reliability was assessed by computing the average measure intraclass correlation coefficient (ICC) using the ICC(2,*k*) form (Shrout and Fleiss, 1979), which corresponds to a two-way random effects model for consistency (McGraw and Wong, 1996). ICC values indicate that inter-rater agreement was high for arousal, ICC(2,30) = 0.92, and tension ratings, ICC(2,30) = 0.91, but only moderate for pleasantness, ICC(2,30) = 0.67. The ICC values obtained for arousal and pleasantness were nearly identical to those reported in Gingras et al. (2014). Moreover, the mean arousal and pleasantness ratings obtained here were also consistent with those obtained on the same excerpts, but with different participants (Gingras et al., 2014), with a rank correlation of *r*_s_(78) = 0.88 (*p* < 0.001) for arousal, and a slightly weaker correlation of *r*_s_(78) = 0.74 (*p* < 0.001) for pleasantness between both experimental groups (Spearman’s correlation coefficient was used because the distribution of the mean arousal ratings deviated significantly from normality as indicated by Shapiro–Wilk’s test, *W* = 0.963, *p* = 0.023, see **Figure 1**).

**Figure 1** shows the two-dimensional emotion spaces corresponding to the set of 80 excerpts, displaying the mean arousal, tension, and pleasantness ratings obtained on each excerpt. Mean pleasantness ratings (range: 3.47–5.30, *M* = 4.41, *SD* = 0.41) exhibited a more restricted range than mean arousal ratings (range: 2.43–6.00, *M* = 3.80, *SD* = 0.82), in line with the ratings reported for the IADS database (Bradley and Lang, 2007) and with other studies using Romantic music (Marin et al., 2012). Mean arousal and mean tension ratings (range: 2.30–5.60, *M* = 3.58, *SD* = 0.76) were highly correlated, with a rank correlation of *r*_s_(78) = 0.93 (*p* < 0.001).

### Characteristics of the Participants in the Pupillary Response Experiment

#### SRS Total Scores

**FIGURE 2:**
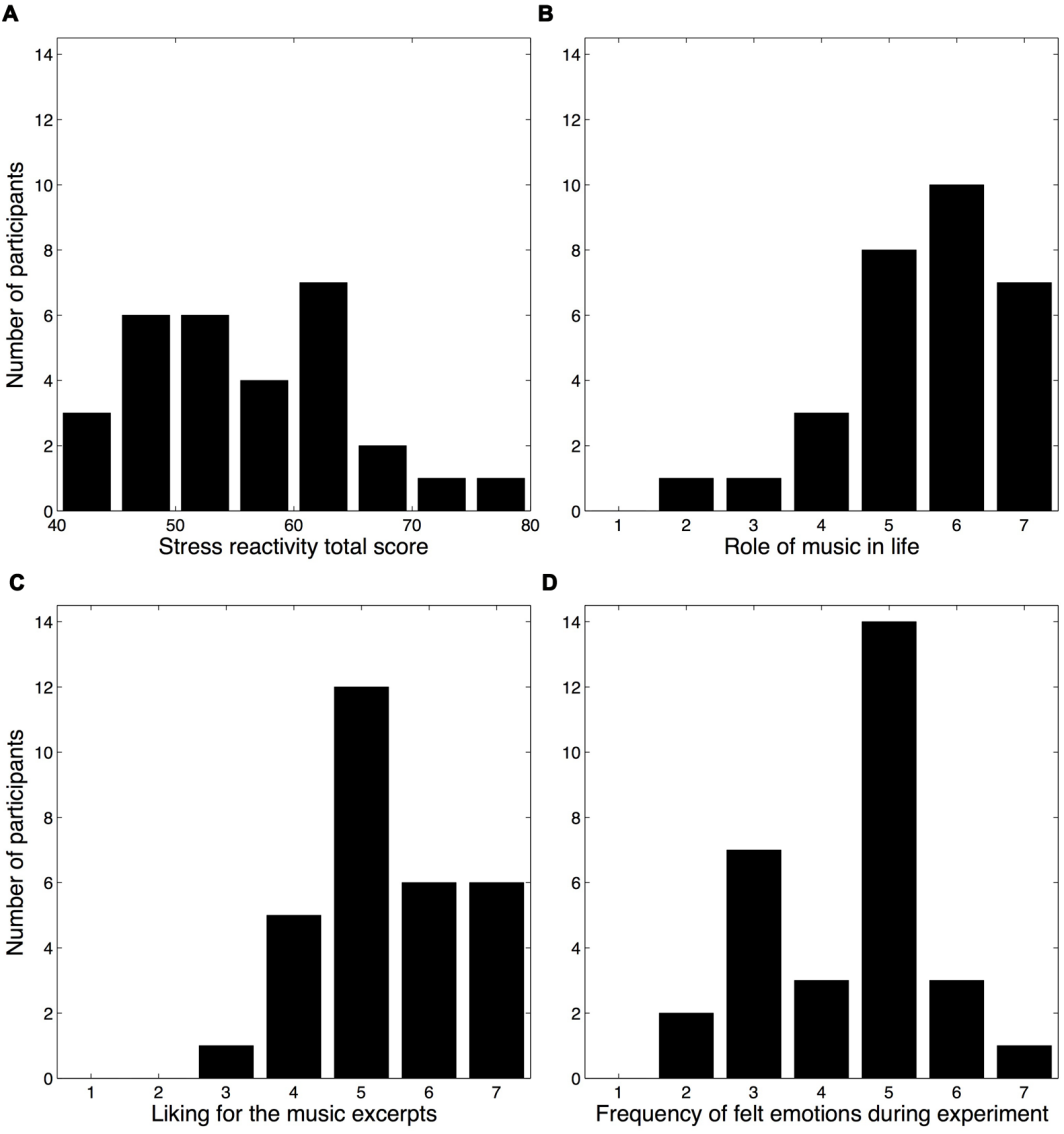
**Histograms of the stress reactivity scores and ratings pertaining to participants’ attitudes toward music. (A)** SRS total scores (Schulz et al., 2005). **(B)** Participants’ ratings regarding the role of music in their life. **(C)** Participants’ overall liking for the music excerpts presented during the experiment. **(D)** Participants’ frequency of felt emotions during the experiment.

Mean SRS total scores (*M* = 55.5, *SD* = 9.0) are shown in **Figure 2A**. Female participants obtained higher scores (*M* = 57.4) than male participants (*M* = 53.6), in line with earlier studies (Nater et al., 2006), but this difference did not reach significance, *t*(14) = 1.21, *p* = 0.25.

#### Attitudes toward Music

Three questions from the post-experiment questionnaire probed the participants’ attitudes toward music, both generally and in regards to the experiment. Although most participants reported that music plays a large role in their life (role of music: *M* = 5.53, *SD* = 1.25), a sizable minority judged its role to be relatively modest (**Figure 2B**). Most participants reported liking the music excerpts, *M* = 5.37, *SD* = 1.10 (**Figure 2C**), whereas the frequency of felt emotions during the experiment exhibited a bimodal distribution (Hartigan’s dip test for unimodality indicated a non-unimodal distribution, *D* = 0.1167, *p* = 0.001), *M* = 4.40, *SD* = 1.25 (**Figure 2D**). No significant differences were observed between male and female participants for any of these three questions (Wilcoxon rank-sum tests, all *p*-values >0.1), nor for the mood subscale scores (MANOVA with gender as between-subjects factor, all *p*-values >0.2).

### Pupillary Responses

**FIGURE 3:**
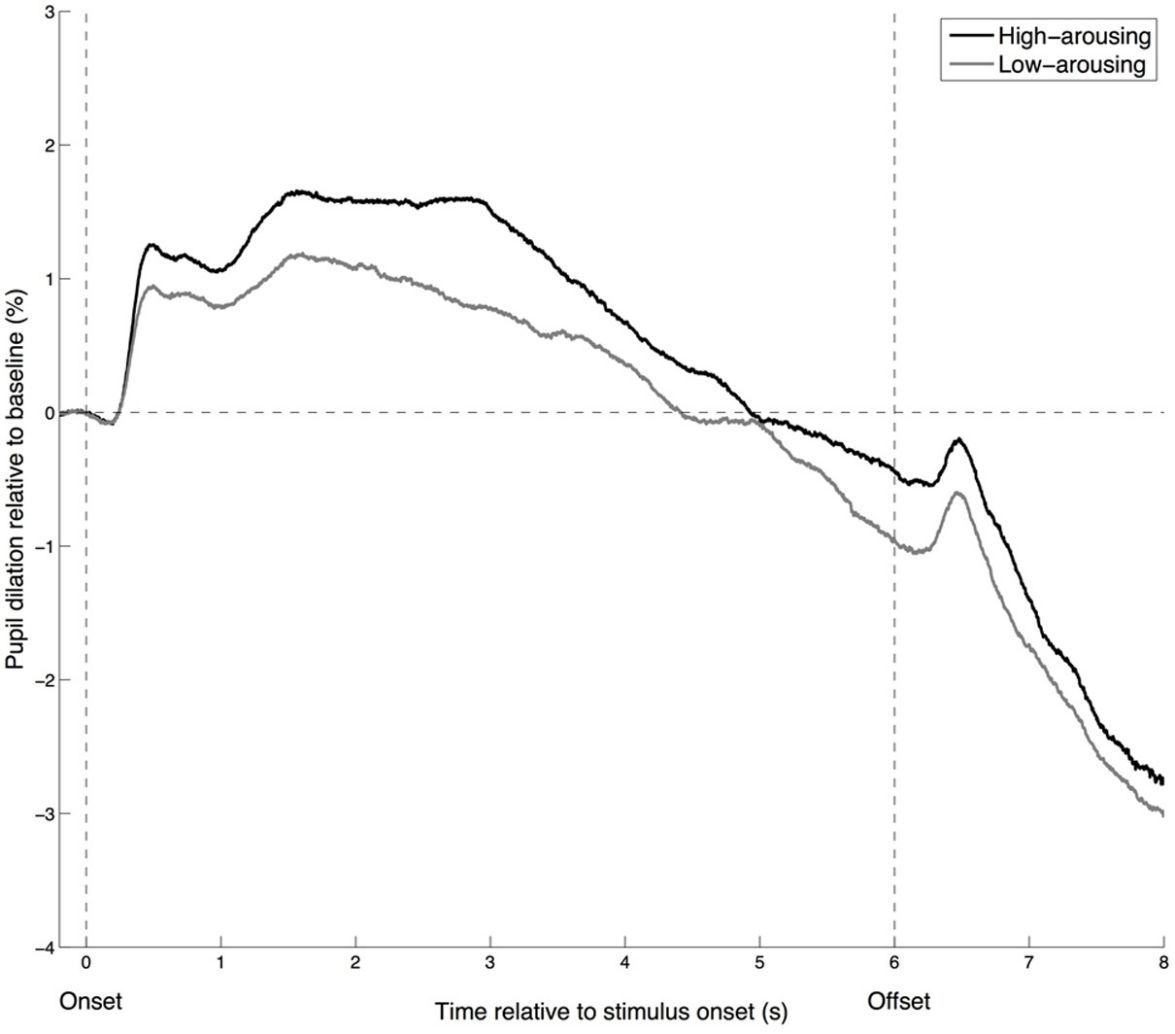
**Time course of the pupillary response for high- and low-arousing excerpts**. Pupil dilation is calculated as a percentage of the mean pupil diameter observed during the 200 ms before the onset (baseline). High-arousing excerpts correspond to the 40 excerpts rated as most arousing, whereas low-arousing excerpts are the 40 rated as least arousing.

To visualize whether the time course of pupillary responses is similar for low- and high-arousing stimuli, we categorized the excerpts into low- and high-arousal brackets. The time course displayed a similar pattern for the 40 excerpts rated as most arousing and the 40 rated as least arousing, although the relative dilation was larger for the high-arousing excerpts (**Figure 3**). A sharp increase in pupil size occurs about 400 ms after the stimulus onset. The peak dilation is reached around 1.5 s after stimulus onset (and maintained for a few seconds for the high-arousing excerpts), followed by a smooth constriction until the stimulus offset. A small dilation occurs 400 ms after the offset, followed by a rapid constriction. These observations are in line with earlier investigations of pupillary responses to affective sounds (Partala and Surakka, 2003).

Because the subjective ratings obtained on the excerpts were retrospective ratings of the entire excerpts, pupillary responses were averaged over the entire 6-s duration of the excerpts (Partala and Surakka, 2003) in order to allow a meaningful investigation of the association between ratings and pupillary responses. As a preliminary analysis of this association, we first computed the correlations between the mean pupillary responses observed for each excerpt and the mean subjective ratings obtained for each excerpt (treating each excerpt as the unit of analysis) on the one hand (**Table 1**), and between the mean pupillary responses observed for each participant and the participant-specific features (i.e., mood subscales, SRS scores and rated attitudes toward music obtained, treating each participant as the unit of analysis) on the other hand (**Table 2**). These analyses showed that mean subjective arousal and tension ratings were positively correlated with the mean pupillary response observed for each excerpt (**Table 1**). Moreover, listeners’ self-reported evaluation of the role of music in their life was significantly correlated with their pupillary responses, averaged over all excerpts (**Table 2**).

**Table 1:** Correlations computed over the mean values obtained for each music excerpt.

	Arousal	Tension	Pleasantness	Familiarity
Arousal				
Tension	0.93^∗∗∗^			
Pleasantness	–0.24^∗^	–0.43^∗∗∗^		
Familiarity	0.16	–0.04	0.40^∗∗∗^	
**Pupillary response**	**0.29^∗∗^**	**0.27^∗^**	**–0.02**	**0.17**

*Rank correlations (Spearman; *df* = 78) are shown because some variables are not normally distributed. ^∗^*p* < 0.05; ^∗∗^*p* < 0.01; ^∗∗∗^*p* < 0.001. The statistical power to detect a large effect size (|*r*| > 0.50) for a sample of this size (*N* = 80) was estimated at 0.998 for two-tailed bivariate correlations with a significance threshold of 0.05*.

**Table 2:** Correlations computed over the values obtained for each participant.

	Positive mood	Alertness	Quietude	SRS scores	Role of music	Liking excerpts	Felt emotions
Positive mood							
Alertness	0.37^∗^						
Quietude	0.62^∗∗∗^	0.43^∗^					
SRS scores	0.32^†^	0.07	0.16				
Role of music	0.06	0.17	–0.19	0.02			
Liking excerpts	0.02	–0.01	0.08	0.09	0.02		
Felt emotions	0.14	0.17	0.19	0.20	–0.20	0.27	
**Pupillary response**	**0.24**	**–0.08**	**0.12**	**0.14**	**0.42^∗^**	**0.05**	**0.06**

*Mean values were used for the pupillary response. Rank correlations (Spearman; *df* = 28) are shown because some variables are not normally distributed. ^∗^*p* < 0.05; ^∗∗^*p* < 0.01; ^∗∗∗^*p* < 0.001; ^†^*p* < 0.10. The statistical power to detect a large effect size (|*r*| > 0.50) for a sample of this size (*N* = 30) was estimated at 0.828 for two-tailed bivariate correlations with a significance threshold of 0.05*.

A more refined analysis combined the subjective ratings associated with each excerpt with the participants’ self-reported mood subscales, SRS scores, and attitudes toward music in a single statistical model to predict the pupillary response for each excerpt and participant. In doing so, we sought to quantify the contribution of excerpt-specific affective characteristics and listener-specific traits to the observed variance in pupillary response among excerpts and participants using maximum-likelihood linear mixed models. Given that each excerpt was heard by each participant, excerpts and participants were treated as fully crossed random effects (Baayen et al., 2008; Judd et al., 2012; Brieber et al., 2014). Here, we began with a full model including all fixed and random effects of interest, and implemented a backward stepwise model selection procedure. Hence, our initial model included arousal, pleasantness, and familiarity ratings as excerpt-specific features (tension ratings were not included to reduce multicollinearity), and gender, mood subscales, SRS scores, and attitudes toward music (role of music, liking for the excerpts, and frequency of felt emotions) as listener-specific features. Additionally, all two-way interactions between each excerpt-specific feature and listener-specific trait were considered (i.e., arousal × gender, pleasantness × SRS scores, etc…). Participant, excerpt, and gender were coded as categorical factors, whereas all other predictors were treated as covariates and grand mean centered (Enders and Tofighi, 2007).

The best-fitting model included the following predictors: listeners’ gender, *b* = –1.31 (with males as the reference category), *SE* = 0.63, χ^2^(1) = 4.29, *p* = 0.038, the reported role of music in their lives, *b* = 0.61, *SE* = 0.25, χ^2^(1) = 5.95, *p* = 0.015, and the arousal ratings of the excerpts, *b* = 0.31, *SE* = 0.11, χ^2^(1) = 8.46*, p* = 0.004. Additionally, a significant interaction between arousal ratings and liking for the excerpts was found, *b* = –0.29, *SE* = 0.10, χ^2^(1) = 8.41*, p* = 0.004, meaning that the pupillary responses of listeners who liked the excerpts greatly were modulated less strongly by arousal than those of listeners who did not like the excerpts as much. The final model also included a random intercept associated with each listener, χ^2^(1) = 203.28*, p* < 0.001, but no random intercept for excerpts as its inclusion did not improve the model fit.

According to the model, males were predicted to show stronger pupillary dilations than females (1.31% on average), whereas each additional unit increment in a listener’s reported role of music in their life predicted an increase of 0.61% in the dilation observed for that listener (across all excerpts). Moreover, each additional unit increment in the mean arousal ratings predicted an increase of 0.31% in the dilation observed for a specific excerpt (across all listeners). However, the effect of arousal was much weaker for listeners who liked the excerpts greatly, with a Spearman correlation coefficient between arousal ratings and pupillary responses of 0.39, *p* < 0.001, for the 18 listeners who gave liking ratings of 5 or less, compared to –0.02 for the 12 listeners who gave ratings of 6 or more.

An analogous model was obtained when predicting pupillary responses using tension ratings instead of arousal ratings, with significant effects of gender, reported role of music, tension ratings, and a significant interaction between tension and overall liking for the excerpts. The coefficients and statistical tests also yielded very similar values to those obtained for the arousal model, which is to be expected considering the very high correlation between arousal and tension ratings.

## Discussion

Pupillary responses to musical stimuli have rarely been investigated. In this study, we collected pupillary responses of non-musicians to a set of 80 six-second music excerpts for which we separately obtained subjective ratings of felt arousal, pleasantness, tension, and familiarity. We hypothesized that arousal ratings of the music excerpts, as well as participants’ attitudes toward music, would predict pupillary responses. A correlational analysis showed that, as predicted, arousal and tension ratings were significantly correlated with mean pupillary response. Among listener-specific characteristics, participants’ reported role of music in their life predicted the magnitude of the pupillary dilation. A linear mixed model analysis including both music- and listener-specific features resulted in a best-fitting model with gender, role of music and arousal ratings as predictors of the pupillary response. Furthermore, an interaction between arousal ratings and liking was found. In general, these results are in line with the hypothesized contribution of excerpt-specific and listener-specific characteristics to pupillary responses to music. However, contrary to our predictions, female participants showed smaller pupillary dilations than males, even though male and female listeners did not significantly differ in their attitude toward music or in their scores on the subscales of the MDBF mood questionnaire. Taken together, these results lend support to models that predict that responses to music depend on characteristics of the listener as well as on the music itself (Hargreaves et al., 2005).

Regarding excerpt-specific features, it is worth noting that pleasantness was not significantly correlated with pupillary responses. This is in agreement with previous reports indicating that pupillary responses are determined by emotional arousal, independently of the perceived pleasantness of the stimuli (Bradley et al., 2008). Furthermore, we note that pleasantness ratings are not as consistent across participants as arousal and tension ratings, and are also more difficult to predict from the acoustical features of the stimuli (Schubert, 2004; Eerola, 2011; Gingras et al., 2014).

Sound intensity, which is one of the main predictors of music-induced subjective arousal, is known to be correlated with physiological responses such as skin conductance (Gomez and Danuser, 2007). However, our findings not only suggest that the range of subjective music-induced arousal ratings is largely unaffected by amplitude normalization (Gingras et al., 2014), but also that physiological responses to music stimuli remain correlated with subjective arousal ratings even in the absence of intensity contrasts between music stimuli.

The relationship between the role of music in participants’ lives and their pupillary responses, as well as the observed interaction between arousal ratings and participants’ liking for the excerpts, are in line with the growing body of literature suggesting that emotional responses to music depend on individual differences (e.g., Liljeström et al., 2013; Mas-Herrero et al., 2013; Park et al., 2013; Mori and Iwanaga, 2015). In contrast to our findings regarding the interaction between arousal ratings and liking for the excerpts, Schäfer and Sedlmeier (2011) reported that skin conductance did not correlate with preference for music, whereas heart and respiration rates did, suggesting that future research will need to further investigate how the preference for music style and autonomic arousal measures are related.

The finding that the role of music in people’s lives predicts their pupillary response to music could be interpreted as an indicator that physiological arousal varies with the level of engagement with music (see Latulipe et al., 2011; Bradshaw et al., 2012). It could be surmised that the importance of music in people’s lives is associated with traits such as absorption as well as with their degree of musical sophistication. Therefore, the relationship between the role of music in people’s lives and physiological responses, such as pupil dilation, may be further investigated by assessing participants’ degree of musical engagement (e.g., Müllensiefen et al., 2014) and absorption (Sandstrom and Russo, 2013) using standardized tests. Furthermore, it should be noted that the questionnaire item regarding the self-reported role of music in participants’ lives did not differentiate between positively or negatively valenced influences, which is an aspect that could be explored in greater detail. More generally, because personality traits, such as neuroticism, have been shown to predict pupillary responses to sound stimuli (Antikainen and Niemi, 1983), future research in this domain should consider the role of personality traits in greater depth.

The larger pupil dilations observed for male listeners stand in contrast to earlier studies reporting stronger psychophysiological, but not psychological, responses to high-arousing, unpleasant music in females compared to males (Nater et al., 2006). This discrepancy with earlier results may be due to the fact that our musical stimuli were not selected to induce high levels of unpleasantness, which is supported by the fact that stress reactivity was not a significant predictor of pupil dilation. Moreover, in contrast to Nater et al. (2006), female participants were not screened for the use of hormonal contraceptives in the present study.

Although we controlled for the potential effect of familiarity by selecting music excerpts from a little-known genre, we observed a positive (but non-significant) correlation between familiarity and pupil dilation. Because the range of familiarity ratings was very restricted, we may suppose that the effect of familiarity and exposure on pupillary responses would be more evident with a set of music excerpts ranging from unfamiliar to very familiar. This is supported by recent findings showing that repeated exposure to unfamiliar music significantly increased skin conductance (a marker of emotional arousal) and that self-reported familiarity ratings were positively related to skin conductance (van den Bosch et al., 2013).

## Conclusion

We show that pupillary responses to music are predicted by a combination of excerpt-specific affective characteristics, such as arousal potential, and listeners’ attitudes toward music. Besides demonstrating that pupil size is a psychophysiological parameter that is sensitive to musically induced emotions and can thus be used to probe listeners’ affective responses to music, our results also point more broadly toward a wide-ranging complementarity between the role of individual differences at the level of music production (Gingras et al., 2013) and at the level of music reception.

## Conflict of Interest Statement

The authors declare that the research was conducted in the absence of any commercial or financial relationships that could be construed as a potential conflict of interest.
