# Supplementary material for: The Eye is Listening: Music-Induced Arousal and Individual Differences Predict Pupillary Responses
Source: Front Hum Neurosci. 2015 Nov 10;9:619. doi: 10.3389/fnhum.2015.00619 (PMC4639616; doi:10.3389/fnhum.2015.00619)
Supplement: Supplementary file 1 [file Appendix.DOCX]

**Appendix: list of musical excerpts**

| No. | Composer | Piece title | Movement | Producer and label |
| --- | --- | --- | --- | --- |
| 01 | Brahms | Trio No. 1 in B maj., Op. 8 | 1 - Allegro con brio | Chandos 8334 |
| 02 | Brahms | Trio No. 1 in B maj., Op. 8 | 2 – Scherzo | Chandos 8334 |
| 03 | Brahms | Trio No. 1 in B maj., Op. 8 | 3 - Adagio | Chandos 8334 |
| 04 | Brahms | Trio No. 1 in B maj., Op. 8 | 4 - Allegro | Chandos 8334 |
| 05 | Brahms | Trio No. 2 in C maj., Op. 87 | 1 - Allegro | Chandos 8334 |
| 06 | Brahms | Trio No. 2 in C maj., Op. 87 | 2 - Andante con moto | Chandos 8334 |
| 07 | Brahms | Trio No. 2 in C maj., Op. 87 | 3 - Scherzo | Chandos 8334 |
| 08 | Brahms | Trio No. 2 in C maj., Op. 87 | 4 - Allegro giocoso | Chandos 8334 |
| 09 | Brahms | Trio No. 3 in C min., Op. 101 | 1 - Allegro energico | Chandos 8334 |
| 10 | Chopin | Trio in G min., Op. 8 | 1 - Allegro con fuoco | Gramola 98934 |
| 11 | Chopin | Trio in G min., Op. 8 | 2 - Scherzo | Gramola 98934 |
| 12 | Chopin | Trio in G min., Op. 8 | 3 - Adagio sostenuto | Gramola 98934 |
| 13 | Chopin | Trio in G min., Op. 8 | 4 - Allegretto | Gramola 98934 |
| 14 | Dvořák | Trio No. 1 in B flat maj., Op. 21 | 1 - Allegro molto | Chandos 241-24 |
| 15 | Dvořák | Trio No. 1 in B flat maj., Op. 21 | 2 - Adagio molto | Chandos 241-24 |
| 16 | Dvořák | Trio No. 1 in B flat maj., Op. 21 | 3 - Allegretto scherzando | Chandos 241-24 |
| 17 | Dvořák | Trio No. 1 in B flat maj., Op. 21 | 4 - Allegro vivace | Chandos 241-24 |
| 18 | Dvořák | Trio No. 2 in G min., Op. 26 | 1 - Allegro moderato | Chandos 241-24 |
| 19 | Dvořák | Trio No. 2 in G min., Op. 26 | 2 - Largo | Chandos 241-24 |
| 20 | Dvořák | Trio No. 2 in G min., Op. 26 | 4 - Allegro non tanto | Chandos 241-24 |
| 21 | Dvořák | Trio No. 3 in F min., Op. 65 | 1 - Allegro | Chandos 241-24 |
| 22 | Dvořák | Trio No. 3 in F min., Op. 65 | 2 - Allegro grazioso | Chandos 241-24 |
| 23* | Dvořák | Trio No. 3 in F min., Op. 65 | 3 - Poco adagio | Chandos 241-24 |
| 24 | Dvořák | Trio No. 3 in F min., Op. 65 | 4 - Allegro con brio | Chandos 241-24 |
| 25 | Dvořák | Trio No. 4 in E min., Op. 90, | 1- Lento maestoso | Chandos 241-24 |
| 26 | Dvořák | Trio No. 4 in E min., Op. 90 | 2 - Poco adagio | Chandos 241-24 |
| 27 | Dvořák | Trio No. 4 in E min., Op. 90 | 3 - Andante | Chandos 241-24 |
| 28 | Dvořák | Trio No. 4 in E min., Op. 90, | 4- Allegro | Chandos 241-24 |
| 29 | Dvořák | Trio No. 4 in E min., Op. 90 | 6 - Lento maestoso | Chandos 241-24 |
| 30 | Liszt | Trio in E maj. | N/A | EMI 749979 |
| 31 | Liszt | Tristia - La vallée d'Obermann | N/A | Gramola 98934 |
| 32 | Mendelssohn | Trio No. 1 in D min., Op. 49 | 1 - Molto allegro agitato | Challenge Classics 72097 |
| 33 | Mendelssohn | Trio No. 1 in D min., Op. 49 | 2 - Andante | Challenge Classics 72097 |
| 34 | Mendelssohn | Trio No. 1 in D min., Op. 49 | 4 - Allegro assai | Challenge Classics 72097 |
| 35 | Mendelssohn | Trio No. 2 in C min., Op. 66 | 1 - Allegro energico | Challenge Classics 72097 |
| 36 | Mendelssohn | Trio No. 2 in C min., Op. 66 | 2 - Andante espressivo | Challenge Classics 72097 |
| 37 | Mendelssohn | Trio No. 2 in C min., Op. 66 | 3 - Scherzo | Challenge Classics 72097 |
| 38 | Mendelssohn | Trio No. 2 in C min., Op. 66 | 4 - Allegro appassionato | Challenge Classics 72097 |
| 39 | Saint-Saëns | Trio No. 1 in F maj., Op. 18 | 1 - Allegro | Naxos 8550935 |
| 40 | Saint-Saëns | Trio No. 1 in F maj., Op. 18 | 2 - Andante | Naxos 8550935 |
| 41* | Saint-Saëns | Trio No. 1 in F maj., Op. 18 | 3 - Scherzo | Naxos 8550935 |
| 42 | Saint-Saëns | Trio No. 1 in F maj., Op. 18 | 4 - Allegro | Naxos 8550935 |
| 43 | Saint-Saëns | Trio No. 2 in E min., Op. 92 | 1 - Allegro | Naxos 8550935 |
| 44 | Saint-Saëns | Trio No. 2 in E min., Op. 92 | 3 - Andante | Naxos 8550935 |
| 45 | Saint-Saëns | Trio No. 2 in E min., Op. 92 | 4 - Grazioso | Naxos 8550935 |
| 46 | Saint-Saëns | Trio No. 2 in E min., Op. 92 | 5 - Allegro | Naxos 8550935 |
| 47 | Schubert | Sonatensatz in B flat maj. | 1 - Allegro | Phillips 426096-2 |
| 48 | Schubert | Trio No. 1 in B flat maj., Op. 99 | 1 - Allegro moderato | EMI 749979 |
| 49 | Schubert | Trio No. 1 in B flat maj., Op. 99 | 3 - Scherzo | EMI 749979 |
| 50 | Schubert | Trio No. 1 in B flat maj., Op. 99 | 4 - Allegro vivace | EMI 749979 |
| 51 | Schubert | Trio No. 2 in E flat maj., Op 100 | 1 - Allegro | Phillips 426096-2 |
| 52 | Schubert | Trio No. 2 in E flat maj., Op 100 | 2 - Andante con moto | Phillips 426096-2 |
| 53 | Schubert | Trio No. 2 in E flat maj., Op 100 | 3 - Scherzo | Phillips 426096-2 |
| 54 | Schubert | Trio No. 2 in E flat maj., Op 100 | 4 - Allegro moderato | Phillips 426096-2 |
| 55* | Schumann | 6 Pieces in canon Op. 56 | 3 - Andantino | Challenge Classics 72053 |
| 56 | Schumann | 6 Pieces in canon Op. 56 | 4 - Innig | Challenge Classics 72053 |
| 57 | Schumann | 6 Pieces in canon Op. 56 | 6 - Adagio | Challenge Classics 72053 |
| 58 | Schumann | Trio No. 1 in D min., Op. 63 | 1 - Mit Energie | Hyperion GAW21063 |
| 59 | Schumann | Trio No. 1 in D min., Op. 63 | 2 - Lebhaft | Hyperion GAW21063 |
| 60 | Schumann | Trio No. 1 in D min., Op. 63 | 3 - Langsam | Hyperion GAW21063 |
| 61 | Schumann | Trio No. 1 in D min., Op. 63 | 4 - Mit Feuer | Hyperion GAW21063 |
| 62 | Schumann | Trio No. 2 in F maj., Op. 80 | 1 - Sehr lebhaft | Hyperion GAW21063 |
| 63 | Schumann | Trio No. 2 in F maj., Op. 80 | 2 - Mit innigem Ausdruck | Hyperion GAW21063 |
| 64 | Schumann | Trio No. 2 in F maj., Op. 80 | 3 - In mässiger Bewegung | Hyperion GAW21063 |
| 65 | Schumann | Trio No. 2 in F maj., Op. 80 | 4 - Nicht zu rasch | Hyperion GAW21063 |
| 66 | Schumann | Trio No. 3 in G min., Op. 110 | 2 - Ziemlich langsam | Challenge Classics 72053 |
| 67 | Schumann | Trio No. 3 in G min., Op. 110 | 3 – Rasch | Challenge Classics 72053 |
| 68 | Schumann | Trio No. 3 in G min., Op. 110 | 4 - Kräftig, mit Humor | Challenge Classics 72053 |
| 69 | Smetana | Trio in G min., Op. 15 | 1 - Moderato assai | Dabringhaus 9421512 |
| 70 | Smetana | Trio in G min., Op. 15 | 2 – Allegro, ma non agitato | Dabringhaus 9421512 |
| 71 | Smetana | Trio in G min., Op. 15 | 3 – Presto | Dabringhaus 9421512 |
| 72 | Spohr | Trio No. 1 in E min., Op. 119 | 1 - Allegro moderato | Orfeo 352952 |
| 73 | Spohr | Trio No. 1 in E min., Op. 119 | 3 – Scherzo | Orfeo 352952 |
| 74 | Spohr | Trio No. 2 in F maj., Op. 123 | 1 - Allegro moderato | Orfeo 352952 |
| 75 | Spohr | Trio No. 2 in F maj., Op. 123 | 2 - Larghetto | Orfeo 352952 |
| 76 | Spohr | Trio No. 3 in A min., Op. 124 | 1 - Allegro moderato | Orfeo 352952 |
| 77 | Spohr | Trio No. 3 in A min., Op. 124 | 2 - Andante con Variazioni | Orfeo 352952 |
| 78 | Spohr | Trio No. 3 in A min., Op. 124 | 4 - Presto | Orfeo 352952 |
| 79 | Spohr | Trio No. 4 in B maj., Op. 133 | 3 - Adagio | Orfeo 352952 |
| 80 | Spohr | Trio No. 4 in B maj., Op. 133 | 4 - Presto | Orfeo 352952 |
| 81 | Spohr | Trio No. 5 in G min., Op. 142 | 1 - Allegro vivace | Orfeo 352952 |
| 82 | Spohr | Trio No. 5 in G min., Op. 142 | 2 - Adagio | Orfeo 352952 |
| 83 | Tchaikovsky | Trio in A min., Op. 50 | 1 - Pezzo elegiaco | Dabringhaus 9421512 |
| 84* | Tchaikovsky | Trio in A min., Op. 50 | 3 - Variazione finale e coda | Dabringhaus 9421512 |
| P1† | Brahms | Trio No. 3 in C min., Op. 101 | 4 – Allegro molto | Chandos 8334 |
| P2† | Schumann | 6 Pieces in canon Op. 56 | 5 – Nicht zu schnell | Challenge Classics 72053 |
| P3† | Spohr | Trio No. 3 in A min., Op. 124 | 3 – Scherzo | Orfeo 352952 |

* Excerpts marked with an asterisk obtained mean familiarity ratings above 4 (see Gingras, Marin, & Fitch, 2014) and were not used in the present study.

† Practice excerpts (not included in the actual stimulus set).
